# Use of Long-Acting Injectables for Severe Mental Illness in the Peripartum Period: A Case Report and a Scoping Review

**DOI:** 10.7759/cureus.71378

**Published:** 2024-10-13

**Authors:** Adela-Georgiana Buciuc, Sean E Oldak, Edmi Y Cortes

**Affiliations:** 1 Psychiatry and Behavioral Sciences, University of Miami Miller School of Medicine, Jackson Memorial Hospital, Miami, USA

**Keywords:** acute mania, lai, long-acting injectables, medication nonadherence, peripartum psychosis, pregnancy, substance use obstetrics

## Abstract

The management of acute mania during pregnancy poses a complex clinical task, necessitating careful consideration of treatment options and demanding a delicate balance between the risks associated with medication use and the adverse impacts of untreated severe mental illness on the fetus. Medication nonadherence stands out as a significant factor contributing to relapse, with rates potentially reaching 40%. The pharmacokinetic profile of long-acting injectable (LAI) risperidone contrasts with that of oral risperidone, characterized by a gradual and consistent release from the depot, mitigating fluctuations between peak and trough concentrations. Clinically, this sustained plasma profile of LAI risperidone has been linked to a reduction in adverse events, such as extrapyramidal side effects, metabolic syndrome, and hyperprolactinemia. Numerous studies have indicated that LAI antipsychotic therapy correlates with reduced mortality rates and decreased number of hospitalizations. This case report illustrates the effective management of acute mania in a pregnant 32-year-old through the utilization of LAI risperidone. This case underscores the significance of individualized treatment strategies and emphasizes the potential utility of LAI antipsychotics as a viable therapeutic option for managing acute mania in pregnancy. Further research is warranted to delineate the long-term outcomes and safety profile of LAI antipsychotics in this population.

## Introduction

Managing severe mental illness during pregnancy presents unique challenges, particularly in ensuring medication adherence and fetal safety. Long-acting injectable (LAI) antipsychotics have emerged as a promising option to maintain therapeutic levels, reduce relapse risk, and minimize fluctuations in drug concentrations. Since their introduction in the 1960s, LAI antipsychotics have revolutionized the management of schizophrenia and bipolar disorder. Current guidelines advocate promoting their use early in the course of these illnesses to reduce the risk of relapse, rehospitalization, and mortality rates [1]. The perinatal period is a particularly vulnerable period for the onset and recurrence of psychiatric illness, and untreated psychiatric conditions during pregnancy pose significant risks to both the mother and fetus. This article presents a case report and a brief review of the literature on the use of LAIs in pregnant women with severe mental illness.

## Case presentation

A 32-year-old female, gravida 3 para 2 (G3P2), with a history of bipolar disorder type I with recurrent severe manic episodes with psychotic features and co-occurring severe cannabis use disorder, was admitted following aggressive and disruptive behavior in the community. The patient presented to the emergency department (ED) with disorganized behavior after she was found half-naked wandering the streets. She presented with psychomotor agitation, irritability, labile affect, pressured speech, and tangential thought processes. Thought content was remarkable for grandiose and persecutory delusions with poor attention, distractibility, and lack of insight into the nature of her illness and need for treatment. The patient was found incompetent to provide express and informed consent for voluntary admission and thus incompetent to provide express and informed consent to treatment. She was admitted under involuntary status with her mother signing as a healthcare proxy (HCP). During the initial evaluation, the patient presented with normal vital signs. Laboratory results were unremarkable, except for a positive urine toxicology for tetrahydrocannabinol (THC) and a positive qualitative urine pregnancy test. Initial obstetrical evaluation in the ED estimated gestational age of six weeks on ultrasound and a subsequent beta-human chorionic gonadotropin (beta-HCG) level of 2,345 mIU/ml, which fell within the typical range of 158−31,795 mIU/ml for six weeks of gestation. The patient’s family reported a longstanding history of medication nonadherence but a good prior response to LAI risperidone. Due to administrative issues with her insurance policy, the patient was lost to follow-up in the outpatient setting.

On admission, the patient was initiated on oral risperidone, titrated to 5 mg daily. She required multiple emergency treatment orders (ETOs) and physical restraints during the first few days of hospitalization due to aggressive behavior toward medical staff but showed significant clinical improvement after that. The patient agreed to be discharged to a substance use residential treatment and a psychiatric follow-up in the outpatient setting.

Seven days after discharge, the patient presented again to the emergency department under involuntary psychiatric hold due to aggressive and disruptive behavior in the community. She was re-admitted under involuntary status, with her mother again serving as HCP. Vital signs were stable and within normal limits, and laboratory results were within normal limits except for positive urine toxicology for THC and a beta-HCG of 8,111 mIU/ml. The patient reported that she left the substance use residential facility the same day of discharge from the hospital, was nonadherent to her risperidone treatment, continued the cannabis use, and was unsheltered. The second hospital admission course was similar to the initial one, with multiple ETOs and physical restraints being required for aggressive behavior toward medical staff and other patients. After extensive risk versus benefit discussion and informed consent from the patient’s mother, the patient was initiated on LAI risperidone 120 mg.

At the one-month outpatient follow-up, she showed significant clinical improvement and was compliant with the medication regimen. Additionally, the patient was monitored by obstetricians in the outpatient setting for gestational diabetes and gestational hypertension, with close fetal health assessments.

Literature review

**Figure 1:**
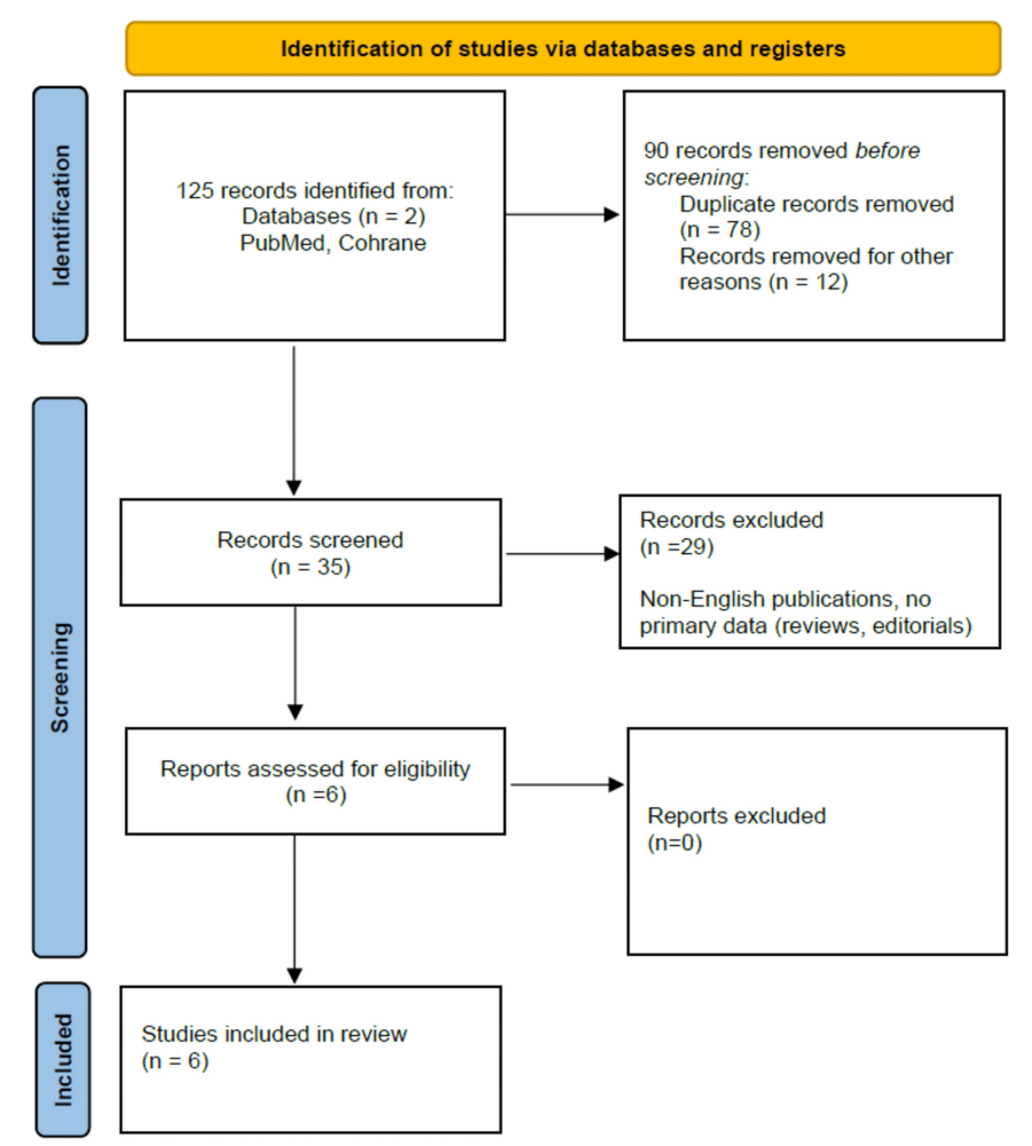
A PRISMA flowchart outlining the study selection process Preferred Reporting Items for Systematic Reviews and Meta-Analyses (PRISMA) Reference: [2]

A comprehensive literature search was conducted using PubMed and Cochrane Library databases. Keywords included “long-acting injectable antipsychotics,” “pregnancy,” “depot antipsychotics,” and specific drug names such as “risperidone LAI,” “paliperidone LAI,” “aripiprazole LAI,” “haloperidol LAI,” and “fluphenazine LAI”. Inclusion criteria comprised studies published in English from 2000 to 2023 that provided data on maternal and fetal outcomes. Exclusion criteria consisted of non-English publications and studies without primary data (reviews, editorials). A total of 125 articles were identified. After screening titles and abstracts, 35 articles were selected for full-text review. Finally, six studies met the inclusion criteria. Figure 1 presents more detailed information on methods. Table 1 showcases a summary of the included studies.

**Table 1:** An overview of the included studies LAI: long-acting injectible

Serial No..	Study	Title and year published	Journal	Sample size	LAIs used	Outcomes
1.	Sung Wan et al. [3]	Use of long-acting injectable risperidone before and throughout pregnancy in schizophrenia (2007)	Progress in Neuro-Psychopharmacology and Biological Psychiatry	1	Risperidone LAI	No adverse outcomes reported
2.	Orsolini et al. [4]	Severe and persistent mental illness (SPMI) in pregnancy and breastfeeding: focus on second-generation long-acting injectable antipsychotics (2021)	Expert Opinion on Drug Safety	11	Five patients received aripiprazole; one patient received olanzapine; three patients received risperidone; two patients received paliperidone	Three preterm vaginal deliveries
3.	Fernandez- Abascal et al. [5]	Long-acting injectable aripiprazole in pregnant women with schizophrenia: a case-series report (2021)	Therapeutic Advances in Psychopharmacology	6	Aripiprazole; one woman interrupted LAI treatment at the end of the first trimester	No adverse outcomes reported
4.	Nguyen et al. [6]	Long-acting injectable antipsychotic treatment during pregnancy: outcomes for women at a tertiary maternity hospital (2022)	Psychiatry Research	36	One patient received haloperidol; two patients received fluphenazine; nine patients received flupenthixol; 12 patients received zuclopenthixol; eight patients received aripiprazole; two patients received paliperidone; four patients received risperidone	Six preterm vaginal deliveries
5.	Eleftheriou et al. [1]	Long-acting injectable antipsychotic treatment during pregnancy: a case series (2023)	International Journal of Environmental Research and Public Health	15	Five patients received paliperidone; 10 patients received aripiprazole	two miscarriages; one still-birth; one preterm vaginal delivery
6.	Ballester-Garcia et al. [7]	Use of long-acting injectable aripiprazole before and through pregnancy in bipolar disorder: a case report (2023)	BMC Pharmacology and Toxicology	1	Aripiprazole	No adverse outcomes reported

## Discussion

The management of severe mental illness during pregnancy is fraught with complexities when balancing the mental health needs of the mother with the safety of the developing fetus. Long-acting injectable antipsychotics offer a viable option to address some of these challenges, primarily by ensuring better adherence and more stable plasma drug levels compared to oral medications. Both first-generation antipsychotics (FGAs) and second-generation antipsychotics (SGAs) have their own sets of risks and benefits and risks when used in LAI form during pregnancy. While FGAs are effective and carry a higher risk of extrapyramidal side effects (EPS), SGAs are associated with more metabolic side effects. Their use in pregnancy remains under-researched, with a paucity of data that is largely limited to case reports and small case series.

**Figure 2:**
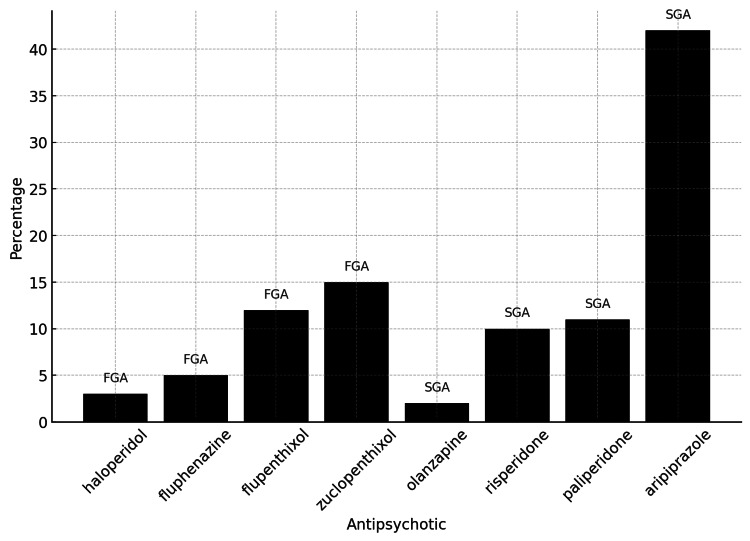
Percentage usage of each antipsychotic among the 69 pregnant women in the studies; the chart provides a clear visual representation of the distribution of both FGAs and SGAs. Sources: [1], [3-7] FGAs: first-generation antipsychotics; SGAs: second-generation antipsychotics

While the initial review covered a total of 70 women from the studies included, one participant from a specific study was excluded from the final analysis due to the discontinuation of LAI treatment at the end of the first trimester. As there was no subsequent monitoring or data available regarding her pregnancy outcomes, she could not be included in the final analysis [5]. Consequently, Table 1 reflects the total sample size at the start of the literature review, whereas the discussion section accounts for the 69 women who continued LAI treatment throughout their pregnancies [1,3-7]. The reported outcomes include preterm vaginal deliveries [1,4,6], miscarriages [1], stillbirths [1], and cases with no adverse outcomes [3,5,7]. The data show that a larger proportion of the women studied used SGA LAIs (34,78%) compared to FGA LAIs (68,12%) [6]. The data indicate a clear preference for SGAs among pregnant women in these studies, reflecting their more favorable side effect profile and broader efficacy [6]. The most used SGA, aripiprazole (64,44% of SGAs), underscores its perceived safety and effectiveness during pregnancy. In contrast, while still significant, the use of FGAs is less prevalent, possibly due to their higher risk of extrapyramidal side effects [6]. Figure 2 shows the percentage usage of each antipsychotic among the 69 pregnant women in the studies, providing a visual representation of the distribution between FGAs and SGAs [1,3-7].

Orsolini et al. reported a 27.27% incidence of preterm deliveries among 11 participants, indicating a significant rate of adverse outcomes [4]. Nguyen et al. reported a 16.67% incidence of preterm deliveries among their larger sample of 36 participants, highlighting the potential risk of preterm birth associated with LAI use [6]. Eleftheriou et al. reported a 26.67% incidence of total adverse outcomes, including preterm deliveries (6.67%), miscarriages (13.33%), and stillbirths (6.67%) among their 15 participants, indicating a higher risk of serious adverse outcomes [7].

General limitations across studies

Firstly, the concomitant use of oral medications alongside LAIs introduces a critical confounding factor. Associating the use of LAIs with oral antipsychotics, mood stabilizers, or antidepressants could confound the results, making it challenging to determine whether adverse outcomes are due to the LAIs or the other medications. Unfortunately, most studies do not provide detailed information on the use of additional medications, which limits the ability to isolate the effects of LAIs.

Additionally, medical comorbidities present another significant source of bias. Participants with psychiatric conditions often have co-occurring medical issues such as diabetes, hypertension, or substance use disorders, which can independently affect pregnancy outcomes. The presence of these comorbidities could lead to adverse events such as preterm delivery, miscarriage, or stillbirth, irrespective of LAI use [4, 5, 8-10]. However, the studies generally do not thoroughly account for these comorbidities, nor do they stratify outcomes based on the presence or severity of these conditions. This oversight can lead to an overestimation or underestimation of the risks associated with LAIs.

The retrospective design of several studies included in our review introduces recall bias, as the accuracy of reported outcomes relies on the participants' memory and medical records, which may not always be comprehensive or accurate. Moreover, the lack of control groups in many studies prevents a direct comparison between those using LAIs and those not using them, further complicating the interpretation of outcomes.

Overall, these potential biases underscore the complexity of studying LAI use during pregnancy. The concomitant use of other medications and the presence of medical comorbidities are significant confounding factors that need to be meticulously accounted for in future research. The inconsistencies in findings underscore the importance of conducting more extensive and methodologically robust studies to provide clearer insights into the effects of LAIs on pregnancy outcomes.

Current understanding and clinical guidelines

The current understanding, based on available evidence and clinical guidelines, supports the use of LAIs in pregnant women with severe psychiatric disorders such as schizophrenia or bipolar disorder, particularly in cases where medication adherence is problematic and/or of clinical concern [6]. The primary advantage of LAIs lies in their ability to provide consistent medication delivery, which is crucial for preventing relapse in patients with severe mental illness. Given the pharmacokinetic changes during pregnancy, such as increased blood volume and body mass, these medications can help maintain therapeutic drug levels more effectively than oral formulations.

Available data regarding outcomes are limited and conflicting. The studies included in our literature review have documented various outcomes, including preterm deliveries, miscarriages, and stillbirths, but these findings are often constrained by the above-mentioned study limitations, which complicate the attribution association of adverse outcomes directly to LAIs.

First-generation antipsychotics vs. second-generation antipsychotics

First-generation antipsychotics, or typical antipsychotics, primarily act as dopamine D2 receptor antagonists. Common FGAs available in LAI form include haloperidol and fluphenazine. These medications have been used for decades, providing substantial clinical experience regarding their efficacy and safety profiles. First-generation antipsychotics are effective in controlling positive symptoms of schizophrenia, though they are less effective against negative symptoms and cognitive deficits. Despite this, FGAs are associated with a higher risk of EPS, including tardive dyskinesia, dystonia, and parkinsonism, which can significantly impact the quality of life [7,9,10].

Regarding safety, the teratogenic risk data for FGAs is relatively limited but generally suggests a low risk of major congenital malformations. Some studies have indicated potential risks for neonatal EPS [6,9,10] and withdrawal symptoms if used during pregnancy [6,9,10]. Clinically, FGAs in LAI form can be considered for pregnant patients who have achieved good control of symptoms with these medications before and are at high risk of relapse if switched to an oral form. Monitoring for EPS is crucial, and dose adjustments may be necessary during pregnancy due to altered pharmacokinetics.

Second-generation antipsychotics have a broader mechanism of action, including serotonin 5-HT2A receptor antagonism in addition to dopamine D2 receptor antagonism. They are associated with a lower risk of EPS compared to FGAs [4]. The safety data on SGAs during pregnancy is more robust than for FGAs but still limited. Some studies have reported associations with gestational diabetes, weight gain, and metabolic syndrome, which can complicate pregnancy [4,6,9]. Potential risks for fetal exposure to SGAs observed in the studies included are low birth weight and preterm birth [4,8], though these findings are not consistent across all studies. Monitoring for metabolic side effects is important, especially given the increased risk of gestational diabetes and weight gain. Dose adjustments may be necessary to account for the physiological changes during pregnancy.

Clinical guidelines recommend a careful, individualized approach to the use of LAIs during pregnancy. A thorough risk-benefit analysis should be conducted, considering the severity of the psychiatric condition, the patient's history of medication adherence, and previous pregnancy outcomes. Women of childbearing age who are given LAIs should receive preconception counseling to discuss the potential risks and benefits of continuing these medications during pregnancy and to explore alternative treatments if necessary. If the decision is made to continue a LAI during pregnancy, it is advised that clinicians should use the lowest effective dose to minimize fetal exposure, with close monitoring of both maternal and fetal health to detect any potential complications as early as possible [4,10]. Regular follow-up appointments are recommended to monitor psychiatric symptoms and pregnancy-related issues, necessitating coordination between psychiatrists, obstetricians, and pediatricians.

Pharmacokinetics and fetal exposure

One of the critical considerations in using LAIs during pregnancy is their pharmacokinetic profile. LAIs avoid first-pass metabolism, leading to more predictable and stable drug concentrations, thereby potentially reducing fetal exposure to peak drug levels. This stability is associated with a decrease in adverse events, including extrapyramidal symptoms, metabolic syndrome, and hyperprolactinemia [9], which are concerns with fluctuating drug levels seen with oral medications [4].

Risk-benefit analysis and informed consent

A thorough risk-benefit analysis is imperative when considering LAIs for pregnant patients. Untreated severe mental illness poses significant risks, including self-neglect and engaging in risky and dangerous behaviors such as substance use [10]. Conversely, the use of antipsychotics, including LAIs, must be carefully evaluated for potential teratogenic effects, risks of metabolic complications, low birth weight, and preterm delivery [4,5]. In the case study presented above, the use of risperidone LAI demonstrated the potential for clinical improvement and adherence in a pregnant patient with severe bipolar disorder and psychotic features. Regular monitoring for gestational diabetes and hypertension, along with fetal health assessments, were integral to the management plan. The patient’s positive outcome underscores the potential for LAIs to provide stable psychiatric management during pregnancy.

## Conclusions

The management of severe mental illness during pregnancy presents significant complexities, and the use of LAI antipsychotics can be pivotal in maintaining medication adherence and reducing the risk of relapse. Current guidelines emphasize the importance of preconception counseling, individualized treatment plans, and close monitoring, all within a multidisciplinary approach to ensure the best outcomes for both mother and child. The decision to use LAI antipsychotics in pregnant women should involve a comprehensive risk-benefit analysis and informed consent, with particular attention to maternal metabolic status and regular fetal monitoring to mitigate potential risks.

Additionally, postpartum psychiatric care is crucial, as the risk of relapse remains high. Continuing LAI treatment postpartum can help stabilize the mother's mental health, ensuring a safer environment for both mother and child. However, there is an urgent need for more extensive research to better understand the safety profiles of LAIs during pregnancy and their long-term effects on both mother and child. This research would provide clearer guidance and inform the development of comprehensive treatment protocols for pregnant women with severe mental illness.
